# Identification of candidate genes for malic acid content in tomato fruits and development of associated SNP markers

**DOI:** 10.3389/fpls.2026.1791734

**Published:** 2026-03-24

**Authors:** Shanshan Wang, Chenyu Wang, Ximeng Wei, Lijuan Qie, Ainong Shi, Qingzhen Yin

**Affiliations:** 1 Institute of Cash Crops, Hebei Academy of Agriculture and Forestry Sciences, Shijiazhuang, China; 2 Department of Horticulture, University of Arkansas, Fayetteville, AR, United States

**Keywords:** candidate gene, GWAS, malic acid, SNP markers, tomato

## Abstract

Malic acid is one of the major organic acids in tomato (*Solanum lycopersicum* L.) fruit and plays a critical role in flavor and overall fruit quality. Identifying genes associated with malic acid accumulation and elucidating their genetic mechanisms can provide valuable insights for tomato quality improvement. In this study, a genome-wide association study (GWAS) was conducted to identify candidate genes and single nucleotide polymorphism (SNP) loci associated with malic acid content in tomato fruit. A natural population consisting of 200 advanced-generation tomato inbred lines was used as the experimental material, and malic acid content was measured at the red-ripening stage. High-quality SNPs obtained through DNA resequencing and variant detection were used for GWAS to identify loci associated with variation in fruit malic acid content. The GWAS identified 90 significant SNP loci, all located on chromosome 6. Linkage disequilibrium (LD) block analysis and candidate gene annotation revealed 17 genes within the associated regions. Based on Pfam and Swiss-Prot database annotation and literature review, five candidate genes, Solyc06g072910.2, Solyc06g072920.3, Solyc06g072950.3, Solyc06g072960.1, and Solyc06g073050.2, were identified as being associated with malic acid content. These genes encode members of the aluminum-activated malate transporter (ALMT) family, ABCB transporter family, and NAC domain-containing proteins. Nine SNP variants were detected within the five candidate genes. Primers were developed for six selected SNP loci, and genotyping was performed using the penta-primer amplification refractory mutation system (PARMS). Correlation analysis between SNP genotypes and malic acid phenotypes demonstrated a strong association, confirming that these loci are significantly related to malic acid content in tomato fruit. The molecular markers developed in this study provide valuable tools for marker-assisted selection, and the identified candidate genes establish a foundation for further investigation into malic acid biosynthesis and metabolic regulation in tomato.

## 1 Introduction

Tomato (*Solanum lycopersicum*), belonging to the Solanaceae family, is a typical annual herbaceous crop. Due to its broad ecological adaptability and economic value, it has been cultivated on a large scale in numerous regions worldwide (Razifard et al., 2020). For fleshy fruits, among the various organic acids contributing to sourness, malic acid is particularly crucial. It largely determines the intensity of acidity in the flesh, thereby influencing consumer preferences (Harker et al., 2002; Petkovsek et al., 2007). As a pivotal node in plant metabolic networks, malic acid serves both as an indispensable intermediate in the tricarboxylic acid cycle (Fernie et al., 2004), and as the primary form of organic acid storage within vacuoles (Berüter, 2004), thereby occupying a central role in energy supply and carbon skeleton allocation.

Beyond metabolic functions, malic acid regulates stomatal movement by altering guard cell osmotic potential to influence opening and closing. In acidic soils, it chelates free aluminum ions, reducing toxicity to mitigate aluminum toxicity (Ligaba et al., 2006). In fruits, malic acid accumulation levels largely depend on the activity and regulation of specific transmembrane transport systems on the tonoplast. This implies that its storage, distribution, and the sour taste during fruit ripening are related to quality formation (Berüter, 2004). Vacuoles serve as the primary organelles for malic acid storage in plant cells. Malic acid enters and exits vacuoles via two main pathways: through specific transporters and channels, and with the assistance of proton pumps that facilitate the transport of organic acids from the cytoplasm into vacuoles (Huang et al., 2021).

Malate transport into vacuoles is catalyzed by at least one transporter and several channels. The *A. thaliana* tonoplast dicarboxylate transporter (*At*tDT) is an essential malate channel (Hurth et al., 2005). *S. lycopersicum* Aluminium-activated malate transporters 11 (*Sl*ALMT11) in tomato is a typical tonoplast anion channel that mediates the anionic transport of malic acid across the vacuole membrane, serving as an important complementary pathway for malic acid transport in tomato (Sasaki et al., 2022). The ALMT gene family plays a major role in malate transport. The S384 residue of wheat *Ta*-ALMT1 (*Triticum aestivum*) modulates protein activity via its phosphorylation level, thereby regulating malate transport in response to varying Al³^+^ concentrations (Sasaki et al., 2004). The *At*ALMT1 transporter on the cell membranes of Arabidopsis root tip cells is responsible for secreting malate extracellularly, where it chelates with soil Al^3+^ to form non-toxic complexes. Simultaneously, it transports ions such as Cl^-^, NO^3-^, SO_4_^2-^ inward, a process crucial for plants to alleviate aluminum toxicity (Kobayashi et al., 2007). Other ALMTs perform similar functions in soybean and alfalfa (Chen et al., 2013; Liang et al., 2013). Among ALMT family members, ALMT9 is the most extensively studied gene, and its genetic variations have been dissected to some extent (Huang et al., 2021; Gai et al., 2023). In apples, the two ALMT9 homologs *Ma1* and *Ma2* in the genomic region Ma (Malic acid) are the primary loci responsible for malic acid accumulation (Bai et al., 2012), The truncation of the conserved domain at the C-terminal of *Ma1* reduces its malic acid transport function (Li et al., 2020). Ye et al. (2017) integrated mGWAS, linkage mapping, and functional analyses to identify a major-effect locus, TFM6 (tomato fruit malate 6), on chromosome 6 that regulates fruit malate accumulation. A 3-bp InDel variation in the promoter region is the key genetic factor responsible for the differences in malic acid content among different tomato germplasm resources. This variation modulates the expression level of *SlALMT9* by affecting the binding efficiency of the *Sl*WRKY42 transcriptional repressor, thereby altering the accumulation of malic acid in tomato fruits (Ye et al., 2017).

In addition to numerous transporters and channels in the plasma membrane, proton pumps are also involved in the transport of malic acid from the cytoplasm to the vacuole (Ma et al., 2019). The regulation of malate transporters and proton pumps relies on complex gene networks, among which transcriptional regulation is the most common and direct method (Huang et al., 2021).Including families such as MYB, bHLH, and WRKY. In apples, *Md*MYB1, *Md*MYB44, and *Md*MYB73 control malic acid accumulation by regulating the transcriptional activity of transporters and proton pumps (Hu et al., 2016). *Md*bHLH3 directly regulates the *Md*cyMDH gene, controlling carbohydrate allocation and malic acid accumulation (Yu et al., 2021). They can also form MYB-bHLH-WD40 (MBW) complexes to act synergistically, enhancing MYB activity and regulating downstream related genes (Xie et al., 2012).Studies on these transcription factors provide an important reference for the gene mapping of malic acid in tomato.

Many previous studies have reported the gene mapping of malic acid in tomato, Fulton et al. (2002) first identified QTL loci associated with soluble acids in tomato using four backcross populations, among which 21 were related to malic acid (Fulton et al., 2002). Sasaki et al. (2016) identified 94 malic acid-associated QTL loci via association analysis, and further studies confirmed that *Sl*ALMT4 and *Sl*ALMT5 are involved in malic acid transport; moreover, the overexpression of *Sl*ALMT5 significantly increased the contents of malic acid and citric acid in the seeds of transgenic tomato (Sasaki et al., 2016). With the advancement of science and technology, genome-wide association studies (GWAS) have identified a number of SNP variants associated with malic acid (Sauvage et al., 2014; Bauchet et al., 2017; Gai et al., 2023). Studies have shown that single nucleotide polymorphisms make a considerable contribution to heritability in malic acid (Zhou et al., 2022). The development of molecular markers based on these SNPs facilitates the acquisition of practical molecular markers that are tightly linked to the malic acid trait.

Malic acid (C_4_H_6_O_5_) is one of the major organic acids in tomato fruit, and its content significantly influences fruit flavor (Qi et al., 2015). Identification of candidate genes controlling malic acid content in tomato fruits is key to elucidating its metabolic pathways, and the development of relevant molecular markers is essential to overcome the current bottleneck of degraded tomato flavor quality and to enable precision breeding. Therefore, this study aimed to (1) identify candidate genes and SNP loci significantly associated with malic acid content in tomato fruits using GWAS, LD block analysis, and multidimensional annotation approaches, and (2) develop practical molecular markers for malic acid content using PARMS technology. These findings provide a theoretical basis for the elucidating the malic acid metabolic network and advancing molecular breeding in tomato.

## 2 Materials and methods

### 2.1 Materials

A total of 200 advanced-generation inbred tomato lines were used as the GWAS panel to identify associated markers, These comprised 171 germplasm from China, 7 from Japan, 6 from USA, 4 from Italy, 3 from Netherlands, 2 from Switzerland, 7 from Israel (**Supplementary Table 1**). 187 genetically independent individual plants were used as an independent validation panel to verify significant loci, detailed information is provided in **Supplementary Table 2**. Seeds for all lines were provided by the Tomato Breeding Laboratory, Institute of Cash Crops, Hebei Academy of Agriculture and Forestry Sciences. The GWAS panel comprised 98 BIG (*S. lycopersicum*), 12 MID (*S. lycopersicum*), and 90 CER (*S. lycopersicum* var.*cerasiforme*) (**Supplementary Table 1**). The validation panel comprised 76 BIG (*S. lycopersicum*), 24 MID (*S. lycopersicum*), and 87 CER (*S. lycopersicum* var.*cerasiforme*) (**Supplementary Table 2**) and was used to validate the SNP loci identified in the GWAS.

### 2.2 Malic acid content measurements

In 2023, the 200 advanced-generation inbred tomato lines in the GWAS panel were grown at the Modern Agricultural Experimental Park, Hebei Academy of Agriculture and Forestry Sciences. Fifteen to twenty plants were grown per advanced-generation inbred line; one plant with vigorous growth, a high fruit set rate, and excellent overall agronomic traits was selected, marked, and assayed for malic acid content. For each selected plant, five to eight cherry tomato fruits with appropriate ripeness and no contamination, and two to four fruits of large- and medium-fruited tomato varieties were harvested. The fruits were chopped, thoroughly mixed, and homogenized with a juicer; each sample was aliquoted into 3 centrifuge tubes, with approximately 100 mg of the homogenate weighed into each tube.

For each sample, the homogenate was aliquoted into three tubes, and approximately 100 mg was weighed into each tube. Calculate the mean values of the malic acid detection results. Malic acid content was determined using a commercial assay kit (Beijing Solarbio Technology Co., Ltd., Beijing, China) following the manufacturer’s instructions. Briefly, samples were mixed thoroughly and incubated at 37 °C for 30 min under light-protected conditions. Absorbance was measured at 450 nm using a SPARK multifunction microplate reader (preheated for 30 min). Readings were recorded as A_assay, A_control, A_standard, and A_blank. Values were calculated as ΔA_assay = A_assay − A_control and ΔA_standard = A_standard − A_blank. A corresponding control tube was included for each assay tube, whereas the blank and standard were measured one to two times per run.

Malic acid content was calculated as follows:


$$\begin{array}{l} \text{Malic acid }(\text{mg}/\text{g})\\ =0.475\times \Delta \text{A}\_\text{assay}/\Delta \text{A}\_\text{standard}\times (134.09/1000)\times \text{F}/\text{W} \end{array}$$

Where W is the sample mass (g) and F is the dilution factor.

In 2024, at the Modern Agricultural Experimental Park, Institute of Economic Crops, Hebei Academy of Agriculture and Forestry Sciences, A total of 187 genetically independent individual plants were selected from a natural population of approximately 800 tomato plants, individually labeled, and fruits with uniform ripeness were harvested from each plant for the determination of malic acid content (**Supplementary Table 2**). The determination method was the same as above.

### 2.3 Normality test, ANOVA and broad-sense heritability for phenotypic data

Normality test, analysis of variance (ANOVA) and broad-sense heritability estimation were performed on the phenotypic data of tomato malic acid content. The distribution characteristics, variation degree and phenotypic differentiation of the phenotypic data were analyzed, and extreme values were screened by the ggplot2 package in R software; a five-panel plot of phenotypic distribution was generated to judge the normality of phenotypic data distribution. Analysis of variance was conducted based on the generalized linear model (GLM), and the visualization of phenotypic data distribution was implemented via the “Distribution” function in JMP PRO 17 software. Additionally, broad-sense heritability (H^2^) was estimated following (Holland et al., 2003) formula as:


$${\text{H}}^{2}=100*{\text{ σ}}_{\text{G}}^{2}/(\sum_{\text{G}}^{2}+\sum_{\text{e}}^{2}/\text{r})$$

Where σ^2G^ represents the total genetic variance, σ^2e^ is the residual variance, and r is the number of replications. The estimates for σ^2G^ was obtained as [EMS(G) -Var (Residual)]/r, where EMS(G) and Var (Residual) were extracted from the ANOVA table.

### 2.4 Library construction and sequencing

Young leaves were sampled from the same individual tomato plants used for malic acid determination, stored at -80 °C, ground in liquid nitrogen, and genomic DNA was extracted using the CTAB method for subsequent sequencing (Fulton et al., 1995). DNA quality and quantity were assessed prior to library preparation; samples with a DNA concentration of ≥20 ng/µL and a total volume of ≥30 µL were used for sequencing. After quality control, genomic DNA was fragmented by ultrasonication. Fragments of appropriate size were subjected to end repair, 3′-A tailing, adapter ligation, PCR amplification, and purification. PCR products were subsequently denatured and circularized to generate single-stranded circular DNA. DNA nanoballs (DNBs) were produced by rolling circle amplification and loaded onto patterned array sites of the sequencing chip. Paired-end sequencing was performed on the BGI sequencing platform.

### 2.5 Genotype detection and high-quality SNP screening

After quality control of raw sequencing reads, the clean data was aligned to the tomato reference genome (SL4.0; annotation ITAG4.0) obtained from the Sol Genomics Network (SGN) (https://solgenomics.net/organism/Solanum_lycopersicum/genome). Redundant reads were removed using SAMtools (v1.9). Variant calling was performed using the GATK pipeline, including HaplotypeCaller, CombineGVCFs, and GenotypeGVCFs. Variants were filtered using the following criteria: QUAL < 30, QD < 2.0, MQ < 40, and FS > 60.0, while all other parameters followed GATK default settings. The filtered call format (VCF) files were imported into R, where only SNP loci were retained and genotypes were converted into a numerical matrix (0, 1, 2, NA). Sample heterozygosity was calculated, and samples with abnormal heterozygosity values (mean ± 2 standard deviations) were excluded. The distribution of heterozygosity was visualized using the ggplot2 package. Based on the remaining valid samples, high-quality SNP markers were further filtered using a call rate > 0.80 and a MAF > 0.05.

### 2.6 SNP distribution, population structure and PCA

To visualize the genome-wide distribution of SNPs, the physical positions of all SNPs on the 12 tomato chromosomes were extracted. The genome was divided into consecutive, non-overlapping sliding windows of 1 Mb, and the number of SNPs within each window was calculated. SNP density maps across all chromosomes were generated using the plot function in R, with color gradients representing SNP counts per window to illustrate the abundance and uniformity of genome-wide markers distribution. After quality filtering, population genetic structure was analyzed using ADMIXTURE software (Alexander et al., 2009). Clustering was performed with the number of assumed subpopulations (K) ranging from 1 to 10, and the optimal K value was determined based on cross-validation error. PCA was performed using the smartPCA program implemented in the EIGENSOFT software package (Patterson et al., 2006) based on SNP data. The clustering patterns of accessions were visualized using the first three principal components (PC1-PC3), and the proportion of the total genetic variation explained by each principal component was recorded.

### 2.7 Genome-wide association study and candidate gene identification

All filtered SNPs from the 200 accessions were used for GWAS using mixed linear models implemented in EMMAX (emmax-intel64; http://csg.sph.umich.edu/kang/emmax/download/index.html), FaST-LMM (v2.07; https://github.com/fastlmm/FaST-LMM), and GEMMA (v0.98.1; https://github.com/genetics-statistics/GEMMA). (csg.sph.umich.edu) The genome-wide significance threshold was set at −log10(P) > 5, and SNPs exceeding this threshold were considered significantly associated with the trait. Candidate genes were defined as those located within ±100 kb (100 kb upstream and 100 kb downstream) of each significant SNP. Quantile–quantile (Q–Q) plots were generated using ggplot2 (v3.3.0), and Manhattan plots were produced using the qqman package (v0.1.9; https://cran.r-project.org/package=qqman). (CRAN) Functional annotation of candidate genes was performed using the NR, Swiss-Prot, GO, COG, and KEGG databases to infer potential biological functions.

### 2.8 PARMS primer development and genotyping

Penta-primer Amplification Refractory Mutation System (PARMS) genotyping was performed using FAM and HEX as allele-specific reporter fluorophores and ROX as a reference fluorophore. After PCR, fluorescence signals (FAM, HEX, and ROX) were scanned on a Tecan F200 microplate reader to obtain endpoint fluorescence values for genotype calling. For each SNP, an approximately 200-bp genomic sequence flanking the target site was selected for primer design (**Supplementary Table 3**). Primers were designed using Primer 5.0. Each PARMS marker consisted of two allele-specific forward primers and one universal reverse primer; primer sequences are provided in **Table 1**. PCR was conducted in a 10-µL reaction containing 1 µL template DNA, 5 µL 2× PARMS master mix, 0.15 µL of each marker primer (10 mmol/L), 0.4 µL universal reverse primer (10 mmol/L), and nuclease-free water to volume. PCR cycling conditions were as follows: 94 °C for 15 min; 10 cycles of 94 °C for 30 s and 65 °C for 1 min with the annealing temperature decreasing by 0.8 °C per cycle; followed by 10 cycles of 95 °C for 20 s and 57 °C for 1 min (Yang et al., 2022). Genotypes were called and visualized using SNP Decoder (http://www.snpway.com:8339/), which converts fluorescence intensities into intuitive clustering plots. The adapter sequences were FAM: GAAGGTGACCAAGTTCATGCT and HEX: GAAGGTCGGAGTCAACGGATT.

**Table 1:** Primer sequences for SNP variant sites.

Gene ID	Position	Ref	Alt		Primer Sequence 5’-3’
Solyc06g072920.3	42616354	T	C	Rg	GAAGGTCGGAGTCAACGGATT**TTGGGAAAGATACTGTGTCACTGG**
				Ra	GAAGGTGACCAAGTTCATGCT**GTTGGGAAAGATACTGTGTCACTGA**
				F	GTACAACCAAAAGCTTTCAGATTTG
Solyc06g072910.2	42613870	C	T	Rg	GAAGGTGACCAAGTTCATGCT**GATGTAAAATTCGAAAATTCAAACG**
				Ra	GAAGGTCGGAGTCAACGGATT**GATGTAAAATTCGAAAATTCAAACA**
				F	CAACAAAGGATTCAACAGGGC
Solyc06g072950.3	42629731	T	C	Ra	GAAGGTGACCAAGTTCATGCT**GTTCAAGTCTGAGGATTAAGCAGTTA**
				Rg	GAAGGTCGGAGTCAACGGATT**TCAAGTCTGAGGATTAAGCAGTTG**
				F	AGAGGCTTGGTCCAATGACTC
	42630813	A	G	Rt	GAAGGTGACCAAGTTCATGCT**TGTGGTTGCTTATGCCTGTAAGT**
				Rc	GAAGGTCGGAGTCAACGGATT**GTGGTTGCTTATGCCTGTAAGC**
				F	GATATTGGGGCATTTGATACAGAG
Solyc06g072960.1	42632043	G	T	Rc	GAAGGTGACCAAGTTCATGCT**GATTTGTATATCTGGTGCAGCATAC**
				Ra	GAAGGTCGGAGTCAACGGATT**GATTTGTATATCTGGTGCAGCATAA**
				F	TCAGGAACAAAGAAACTCAACTACC
Solyc06g073050.2	42669696	T	C	Ft	GAAGGTGACCAAGTTCATGCT**CTTGTTTTATACAATTTGCTTCAACT**
				Fc	GAAGGTCGGAGTCAACGGATT**TTGTTTTATACAATTTGCTTCAACC**
				R	TTATATTTGTCATAATTGCGCTCA

Bold nucleotides indicate the SNP allele-specific base used for genotyping.

### 2.9 Plot box plots

Box plots were used to visualize the distribution of malic acid content across genotype classes at each SNP locus. Plots were generated in R using the ggplot2 package (R v3.4.4). For each genotype group, the box spans the interquartile range (IQR; 25th percentile, Q1, to 75th percentile, Q3), and the horizontal line within the box indicates the median. Whiskers extend to the most extreme values within Q1−1.5 × IQR and Q3 + 1.5 × IQR; values outside this range were considered outliers and are shown as individual points. The x-axis indicates genotype classes (FAM, HEX, FAMHEX, and NA), and the y-axis indicates malic acid content.

## 3 Results

### 3.1 Tomato malic acid content statistics

**Figure 1:**
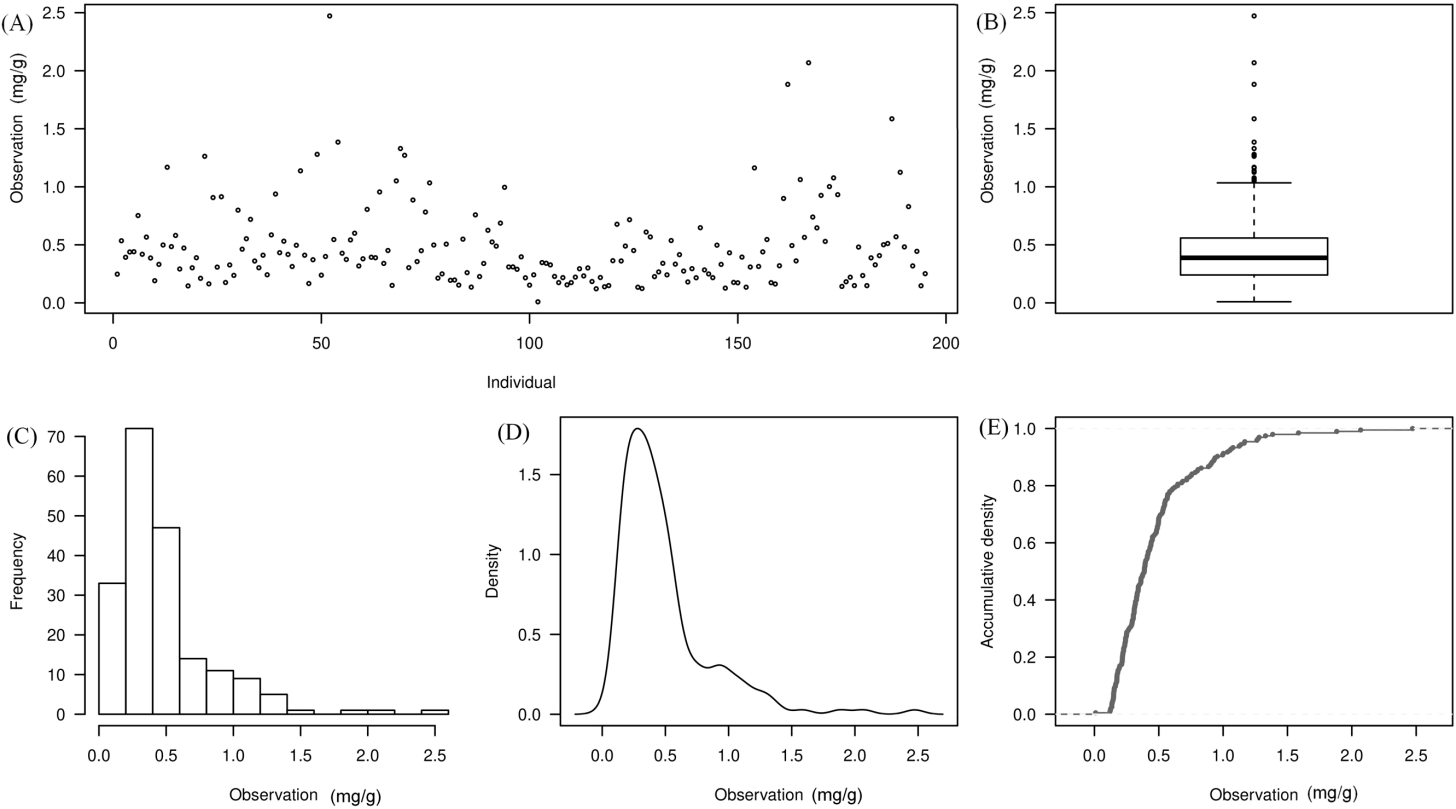
Analysis of 200 tomato malic acid content phenotypic data. **(A)** (Scatter Plot): X-axis represents sample information; Y-axis shows observed phenotypic values (malic acid content); **(B)** (Box Plot): Box plot plotted using phenotypic values (malic acid content); **(C)** (Bar Chart): X-axis representing observed malic acid phenotypic values; Y-axis representing corresponding frequency values; **(D)** (Density plot): X-axis representing observed malic acid phenotypic values; Y-axis representing corresponding frequency values; **(E)** (Cumulative plot): X-axis representing phenotypic malic acid values; Y-axis representing cumulative density values.

Malic acid content was measured in 200 advanced-generation inbred tomato lines for GWAS (**Supplementary Table 1**). Phenotypic data analysis of this population revealed a concentrated distribution in the low-to-moderate value range, with only a few highly significant outliers observed. The overall quality of the phenotypic data was good (**Figures 1A, B**). Descriptive statistics (maximum, minimum, mean, standard deviation, and standard error) are summarized in **Table 2**. After excluding missing values, 195 lines were retained for analysis, with a mean malic acid content of 0.48 mg/g and a standard deviation of 0.36. The median value was 0.36 mg/g. The highest malic acid content was observed in line 502 (2.47 mg/g), whereas the lowest value occurred in line 572 (0.0094 mg/g), indicating substantial phenotypic variation across the panel. The frequency distribution (**Figures 1C, D**) showed that most lines had malic acid content within 0-1.0 mg/g, with frequencies decreasing sharply above 1.0 mg/g, indicating a right-skewed distribution. The cumulative density curve (**Figure 1E**) increased rapidly between 0.0 and 0.5 mg/g and then gradually plateaued, consistent with the concentration of most observations in the 0.0-0.5 mg/g range. The broad-sense heritability for malic acid content was estimated at 64.4% (**Supplementary Table 3**). Overall, the trait exhibited continuous variation suitable for GWAS.

**Table 2:** Statistics on malic acid content in 200tomato high-generation inbred lines.

Number	Mean	SD	Median	Min	Max	Range	Standard error
195	0.4823mg/g	0.36223	0.3880	0.0094	2.47	2.46	0.026

### 3.2 SNP filtering and population structure analysis

**Figure 2:**
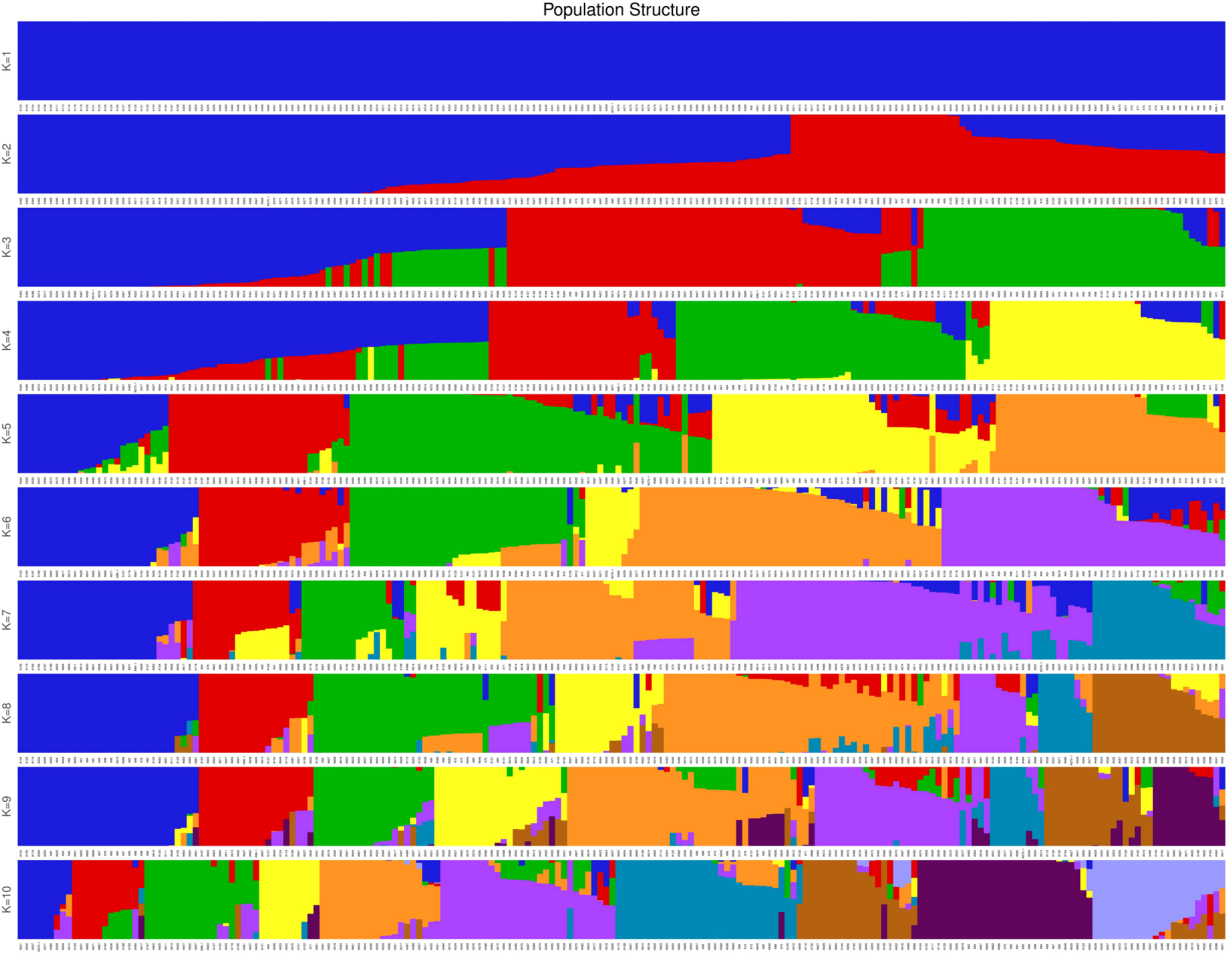
Sample clustering results corresponding to different K values in the admixture analysis.

Based on genotypic data and quality control filtering, a total of 2,991,459 high-quality SNP loci were retained and distributed across the 12 chromosomes of the tomato genome (**Supplementary Figure 1**; **Table 3**). Analysis of individual heterozygosity (**Supplementary Figure 2**; **Supplementary Table 4**) indicated that an average heterozygosity 6.83%, with only a small number of accessions exhibiting heterozygosity levels exceeding 30%. This pattern is consistent with the breeding history of the materials, as most tomato inbred lines derived from multiple generations of selfing are expected to be highly homozygous, whereas a limited number of lines may still be in a segregating state. Population structure was inferred using Admixture software and cross-validation analysis indicated that the optimal number of subpopulations was K = 3, under which the 200 tomato accessions were clearly divided into three genetic groups (**Figure 2**). Principal component analysis (PCA) based on genome-wide SNP data further supported the population structure results. The first three principal components (PC1, PC2, PC3) explained 24.77%, 17.56% and 8.29% of the total variation, respectively (**Supplementary Figure 3**). These three principal components were subsequently included as covariates in the GWAS model to control for population stratification.

**Table 3:** Number of SNPs per chromosome.

Chr	Length(Mb)	SNP number
SL4.0ch00	9.64	16038
SL4.0ch01	90.86	141450
SL4.0ch02	53.47	102046
SL4.0ch03	65.30	71491
SL4.0ch04	64.46	309337
SL4.0ch05	65.27	516288
SL4.0ch06	47.26	405542
SL4.0ch07	67.88	76830
SL4.0ch08	64.00	66095
SL4.0ch09	68.51	689984
SL4.0ch10	64.79	61059
SL4.0ch11	54.38	271209
SL4.0ch12	66.69	264090

### 3.2 Genome-wide association study

**Figure 3:**
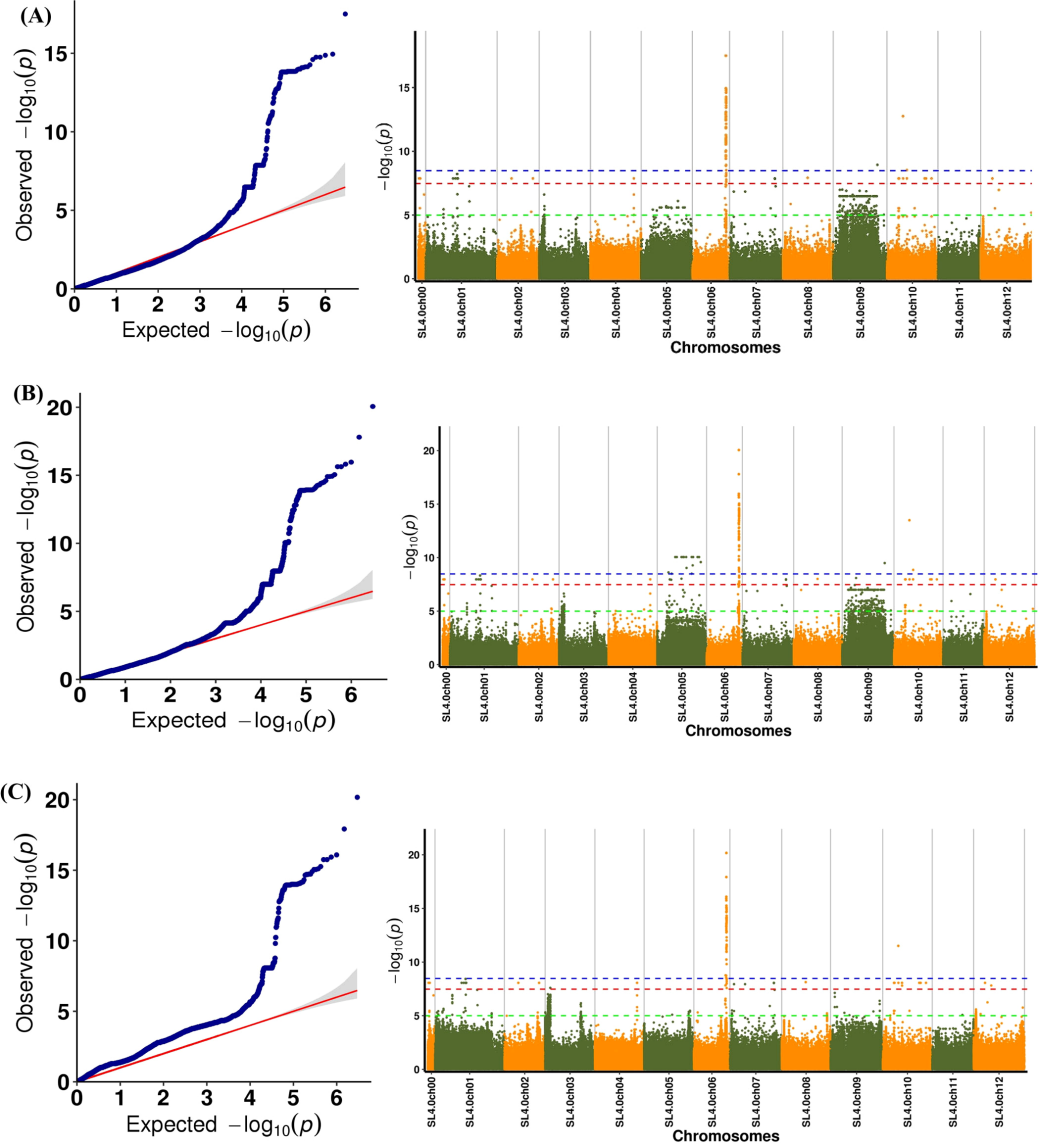
Q-QPlot and Manhattan plot of tomato malate genome-wide association study using **(A)** EMMAX model, **(B)** FaST-LMM model, and **(C)** GEMMA model.

Manhattan and quantile–quantile (Q–Q) plots of the GWAS results for malic acid content were generated using three mixed linear model–based tools (EMMAX, FaST-LMM, and GEMMA). The Manhattan plot summarizes association signals across the genome, whereas the Q–Q plot compares the distribution of observed and expected P-values under the null hypothesis. In the Q–Q plot, the observed values closely followed the reference line at lower significance levels, indicating that most loci conformed to the expected null distribution. In contrast, the upper tail deviated markedly from the reference line and exceeded the confidence interval, suggesting the presence of SNPs with very small P-values and strong association signals for malic acid content. Significant association signals (−log10(P) > 9) were predominantly detected on chromosome 6 (**Figure 3**; **Supplementary Table 5**), indicating that this chromosome likely harbors key loci controlling fruit malic acid content. This pattern is consistent with previous findings reported by Ye et al. (2017), supporting the reliability of the phenotyping and population-based association analysis.

### 3.3 LD block analysis

**Figure 4:**
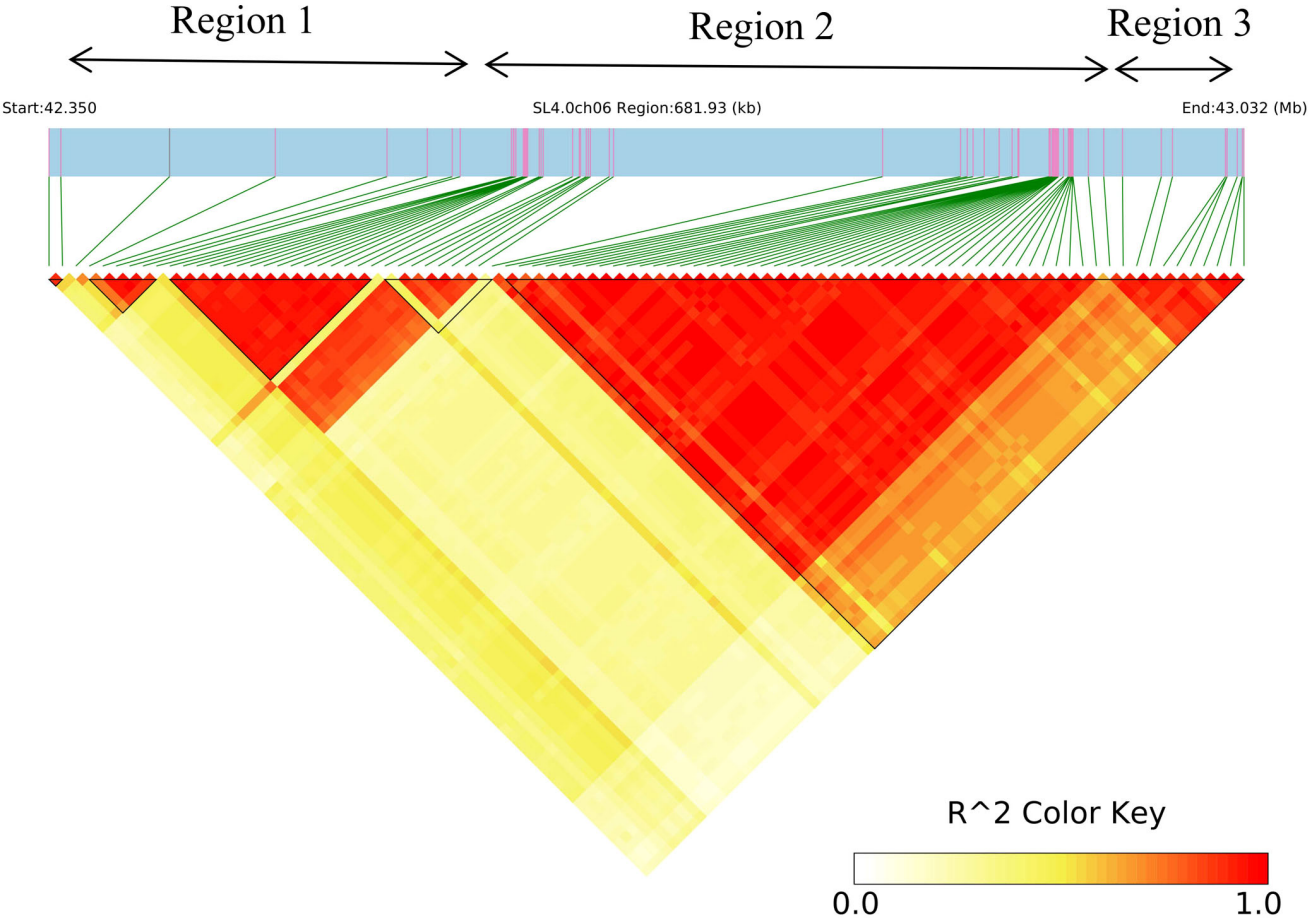
Haploblock showing markers in linkage disequilibrium (LD). R² indicates the strength of linkage disequilibrium, with higher values indicating stronger linkage disequilibrium. Redder colors in the figure denote higher correlations between loci.

Association signals exceeding the genome-wide threshold (−log10(P) > 9) defined a major locus on chromosome 6, spanning 42.3–43.1 Mb from the telomeric end (approximately 0.8 Mb in length). This study aimed to identify more malate-related genes. A Bonferroni-corrected threshold of P < 1/2,991,459 (3.34 × 10^-7^; −log_10_(P) = 6.48) was used for filtration to obtain associated regions, which were then subjected to linkage disequilibrium (LD) block analysis. With this threshold, 162 SNPs were identified from 2,991,459 loci, including 90 SNPs on chromosome 6 (**Supplementary Table 6**). Based on these 90 linked sites, the associated interval on chromosome 6 was broadly partitioned into three LD-defined segments (**Figure 4**): Region 1, 42,349,977–42,672,179 bp; Region 2, 42,870,193–42,934,270 bp; and Region 3, 42,943,041–43,031,911 bp. Single-marker allele frequency comparisons indicated that Regions 2 and 3 showed markedly higher major-allele frequencies than minor-allele frequencies, suggesting weaker genotype–phenotype association signals in these regions (**Table 4**). Therefore, downstream candidate gene identification focused primarily on Region 1.

**Table 4:** Point analysis of SNP loci in different regions.

Description	Region 1	Region 2	Region 3
MAF	0.22-0.24	0.010-0.12	0.065-0.085
Representative Genes	Solyc06g072920.3	Solyc06g073360.3	Solyc06g073520.3
Representative Site	SL4.0ch06:42621045	SL4.0ch06_42870193	SL4.0ch06:42984704
Major Allele	C, 0.7602041	G, 0.905	C, 0.919598
Minor Allele	A, 0.2397959	T, 0.095	T, 0.080402
Allele Type	CC-145, AA-43, AC-8	GG-170,TT-18	CC-182,TT-15,CT-2

### 3.4 Candidate gene and SNP site identification

Region 1 contained 34 SNPs significantly associated with malic acid content. Candidate genes were defined as those located within ±100 kb of each associated SNP, yielding 93 genes in total. Based on COG annotation, these genes were assigned to functional categories including energy production and conversion, carbohydrate transport and metabolism, translation, transcription, post-translational modification, protein transport, signal transduction, and defense mechanisms, with a subset remaining unclassified/unknown (**Supplementary Table 7**).

Because SNPs located within genes may exert more direct effects on trait variation, we further examined variants within gene regions. A total of 33 intragenic SNPs were identified and annotated to 17 genes (**Supplementary Table 8**). Based on Pfam and Swiss-Prot annotations and a literature survey, five genes harboring SNP variants were prioritized as candidates associated with malic acid content: Solyc06g072910.2, Solyc06g072920.3, Solyc06g072950.3, Solyc06g072960.1, and Solyc06g073050.2.

Solyc06g072910.2 and Solyc06g072920.3 were annotated as aluminum-activated malate transporter (ALMT; *Sl*ALMT) genes. Three SNPs were detected in Solyc06g072910.2, including C→T at 42,613,870 bp (synonymous), and G→A at 42,614,975 bp and 42,615,353 bp (located downstream of the coding region). One SNP was detected in Solyc06g072920.3 (T→C at 42,616,354 bp), located upstream of the gene. Solyc06g072950.3 and Solyc06g072960.1 were annotated as ATP-binding cassette (ABC) transporter family (*Sl*ABCB) genes. Two SNPs were identified for Solyc06g072950.3, including T→C at 42,629,731 bp (upstream) and A→G at 42,630,813 bp (downstream). One SNP was detected in Solyc06g072960.1 (G→T at 42,632,043 bp), resulting in a synonymous change. Solyc06g073050.2 was annotated as a NAC transcription factor (*Sl*NACMTF3). Two SNPs were identified, including T→C at 42,669,696 bp (upstream) and A→T at 42,672,179 bp (intron).

**Figure 5:**
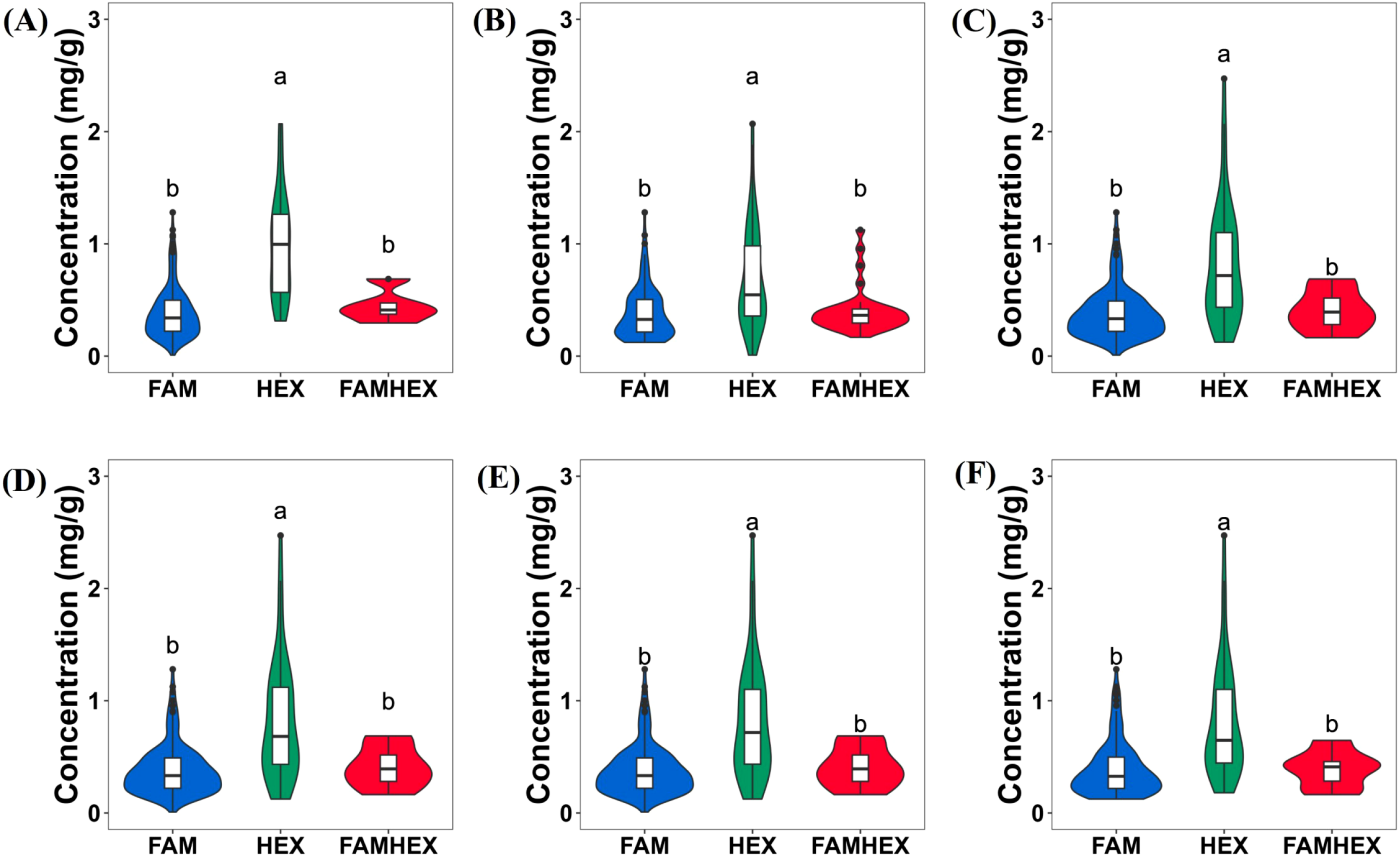
Boxplot of Genotype-Phenotype Correlation at Variant Sites. **(A)** Gene: Solyc06g072910.2 Variant site: 42613870. **(B)** Gene: Solyc06g072920.3 Variant site: 42616354. **(C)** Gene: Solyc06g072950.3 Variant site: 42629731. **(D)** Gene: Solyc06g072950.3 Variant site: 42630813. **(E)** Gene: Solyc06g072960.1 Variant site: 42632043. **(F)** Gene: Solyc06g073050.2 Variant site: 42669696. HEX genotype indicates high malic acid content, FAMHEX genotype indicates moderate malic acid content, and FAM genotype indicates low malic acid content. The same as below.

Based on these candidates, six SNPs were selected for primer design, and PARMS genotyping was conducted in the 200 GWAS panel lines. Boxplot analyses integrating genotype calls with malic acid phenotypes showed that the FAM genotype was associated with lower malic acid content, whereas the HEX genotype was associated with higher malic acid content. In addition, the mutation sites 42,629,731, 42,630,813, and 42,632,043 are closely related with nearly identical detection results, and thus show similar box plots. These results indicate a clear correspondence between genotypes at the six SNP loci and malic acid levels (**Figure 5**; **Supplementary Table 1**).

### 3.5 Validation analysis of candidate SNP sites using PARMS technology

**Figure 6:**
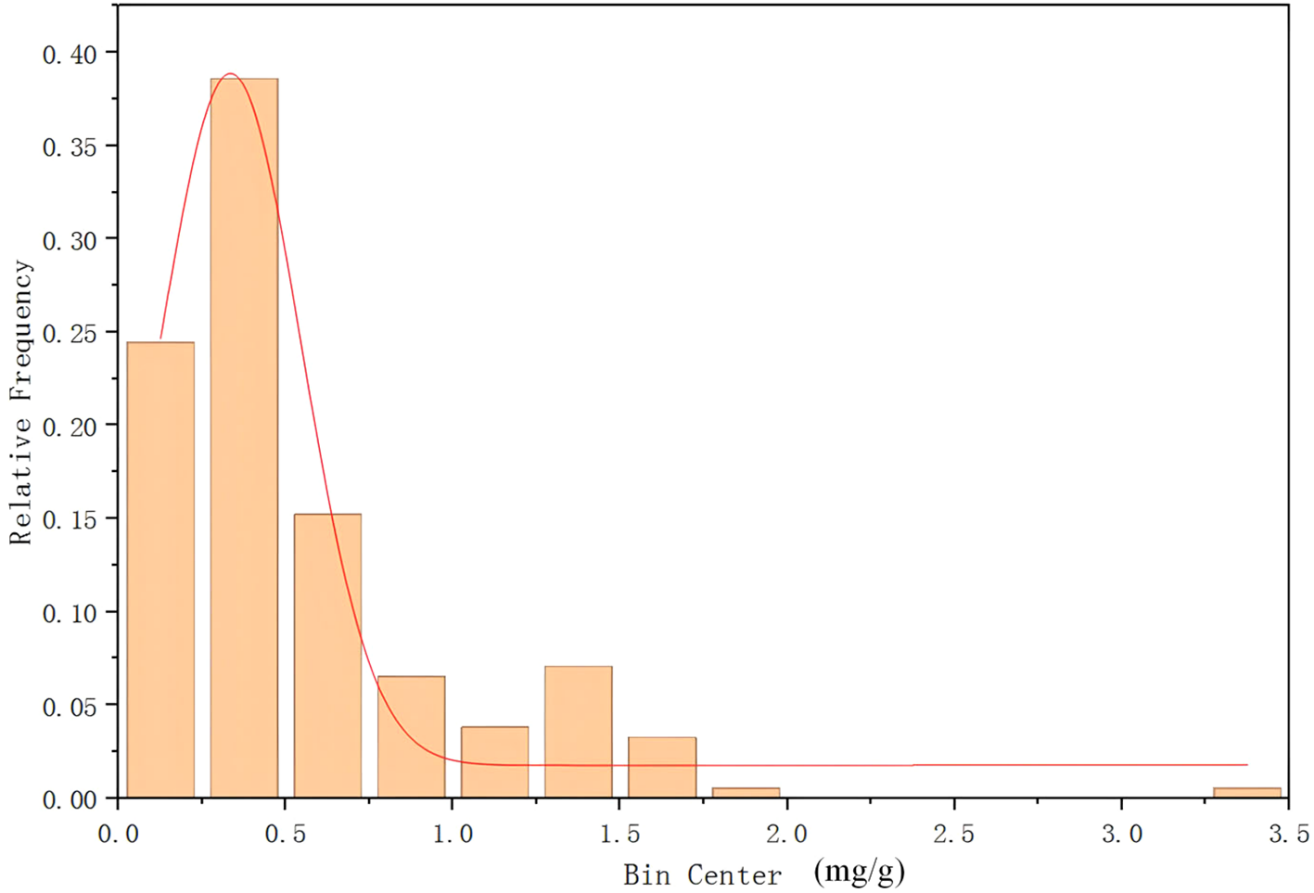
Frequency distribution of malic acid content in 187 common tomato samples.

To validate the association between the six candidate SNP loci and malic acid content, malic acid levels were quantified in an independent panel of 187 tomato accessions (**Supplementary Table 2**). The frequency distribution of malic acid content showed that most accessions clustered within 0-0.8 mg/g, with frequencies decreasing sharply above 0.8 mg/g, indicating a right-skewed distribution (**Figure 6**). Despite this skew, the trait exhibited continuous variation and was suitable for association-based validation.

**Figure 7:**
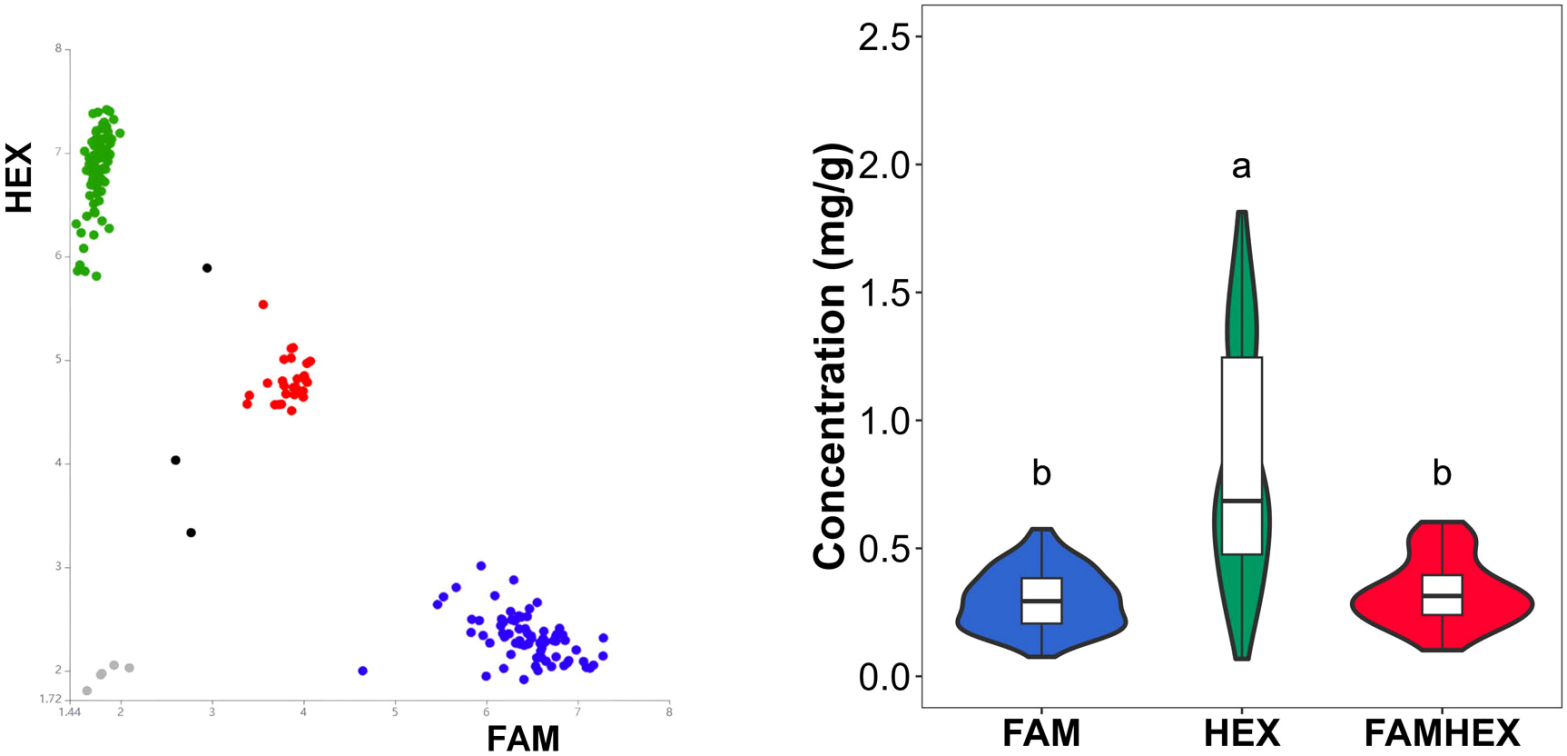
Genotyping and boxplot of the 42616354 variant site in 187 common tomato samples. The green dot in the figure indicates the HEX genotype, the red dot indicates the presence of FAMHEX genotype, the blue dot indicates the presence of FAM genotype, the gray dot indicates the inability to judge, and the black dot indicates the negative control, the same below.

**Figure 8:**
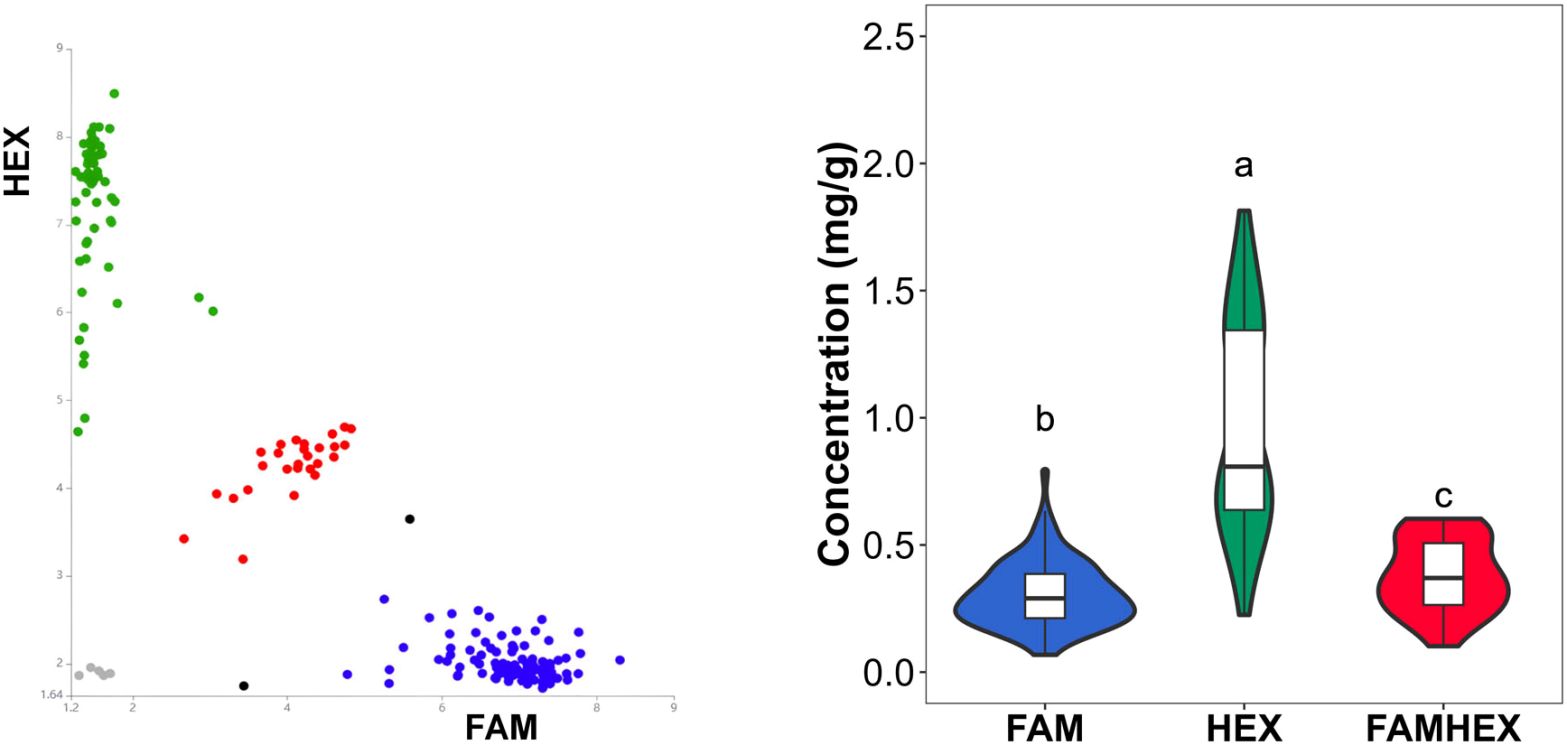
Genotyping map and box plot of the 42613870 variant site in 187 common tomato samples.

**Figure 9:**
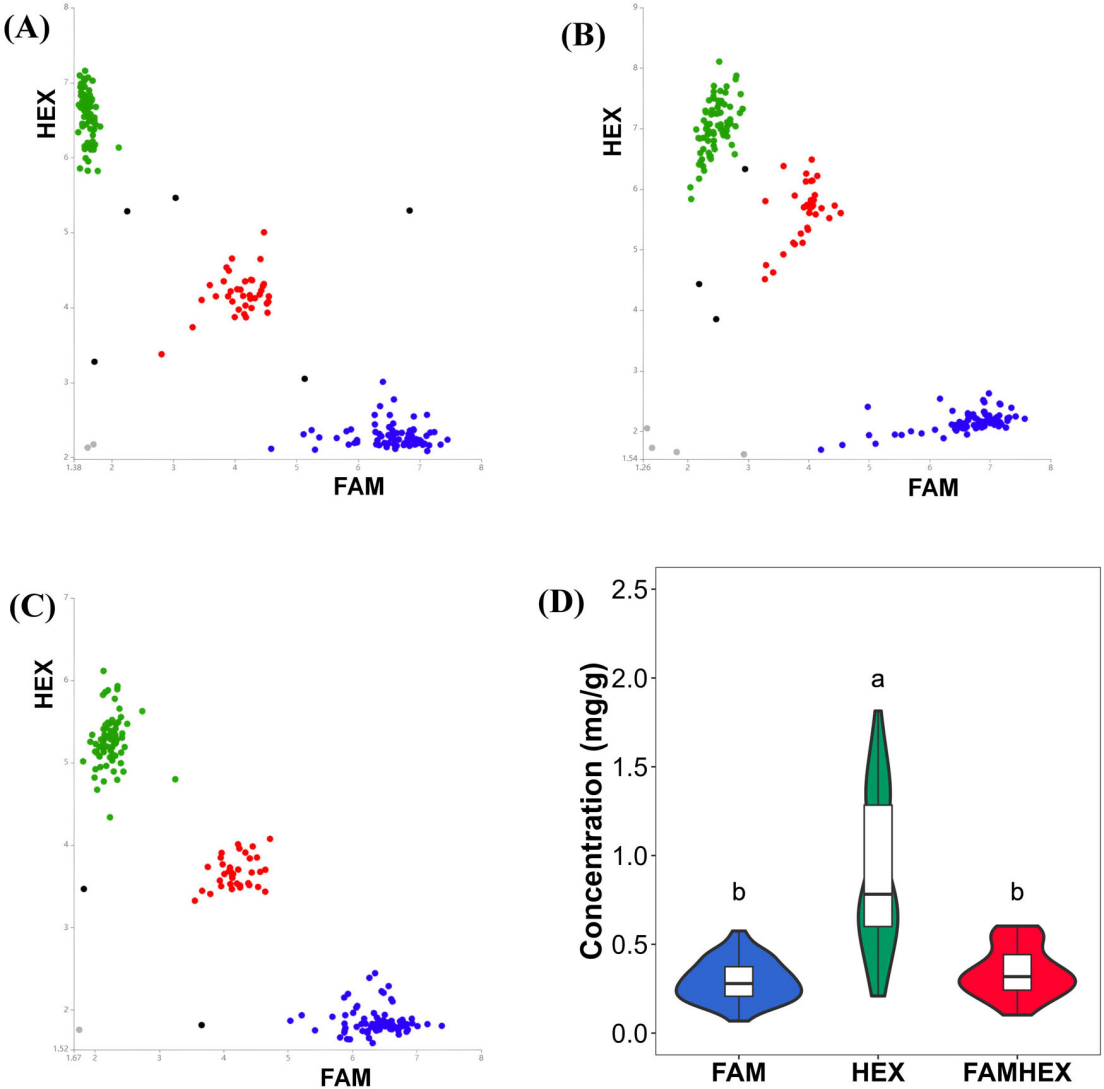
Genotyping and Boxplot **(D)** of variant sites in the *Sl*ABCB Gene Family from 187 Common Tomato samples. **(A)** Gene: Solyc06g072950.3 Variant site: 42629731. **(B)** Gene: Solyc06g072950.3 Variant site: 42630813. **(C)** Gene: Solyc06g072960.1 Variant site: 42632043.

**Figure 10:**
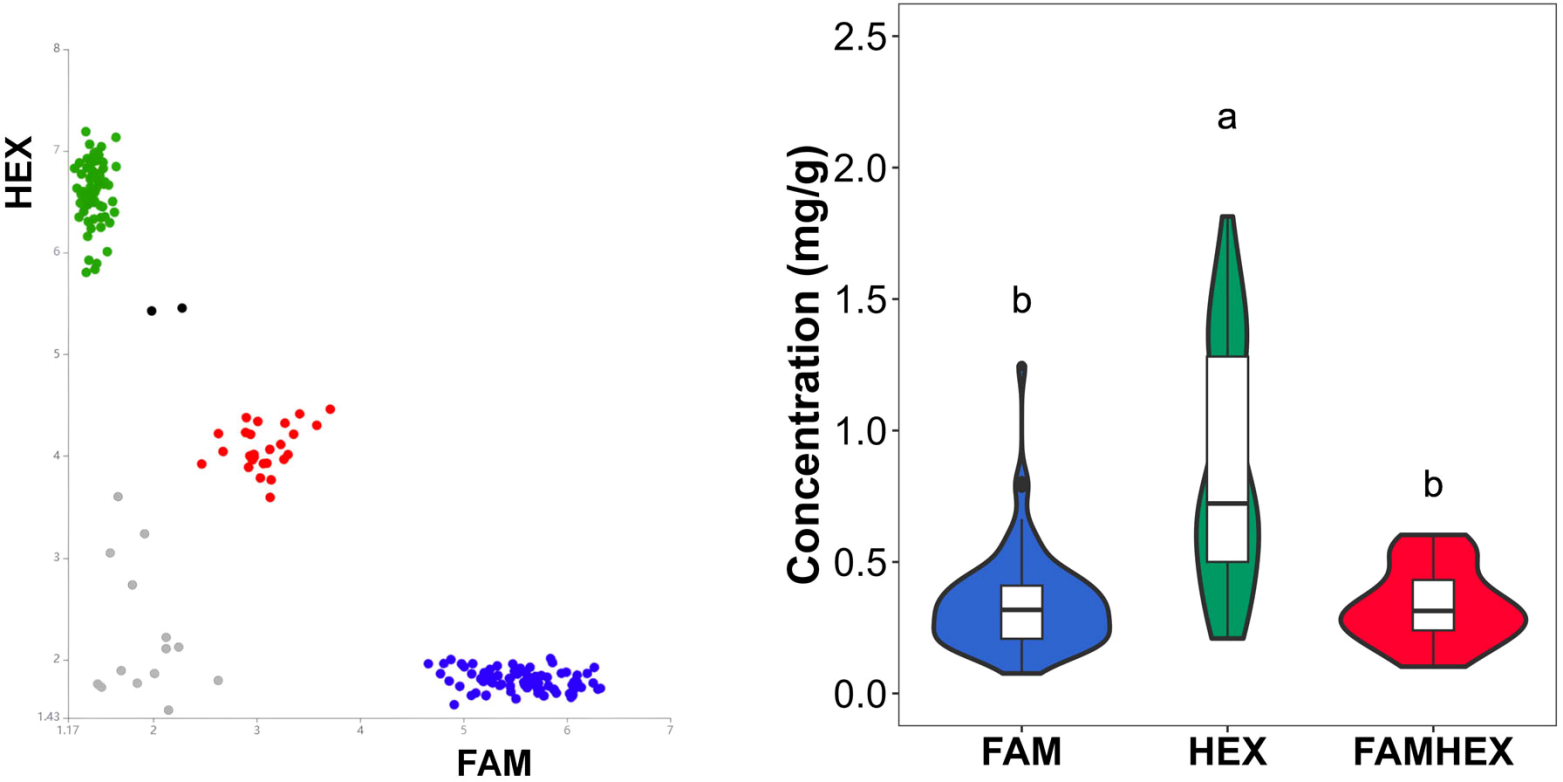
Genotyping and boxplot of variant site 42669696 in 187 common tomato samples.

PARMS genotyping results for the six SNP loci are summarized in **Supplementary Table 2**. Clear genotype clustering patterns were observed (**Figures 7**–**10**), indicating high genotyping quality and a high call rate across loci. Boxplot analyses further showed consistent correspondence between genotype classes at each SNP and malic acid content, supporting the role of SNP variation in the *SlALMT*, *SlABCB*, and *SlNACMTF3* candidate gene families in regulating malic acid accumulation in tomato fruits. In particular, the SNP at 42,613,870 bp in *SlALMT* (Solyc06g072910.2) and three SNPs in *SlABCB* (Solyc06g072950.3 and Solyc06g072960.1) showed pronounced effects, with significant phenotypic differences observed between the FAMHEX and FAM genotype classes.

## 4 Discussion

### 4.1 Localization of the malic acid gene in tomatoes

Genome-wide association studies (GWAS) are widely used to identify loci underlying complex traits and have been applied extensively in crop improvement (Huang et al., 2010; Brachi et al., 2011; Shrestha et al., 2018), including the genetic dissection of yield, stress tolerance, quality-related traits, fruit characteristics and flavor, and disease resistance (Kayikci et al., 2025). Compared with biparental mapping, GWAS leverages natural variation in diverse panels and does not require the development of dedicated mapping populations, enabling more rapid identification of trait-associated SNPs and facilitating marker-assisted breeding.

Substantial progress has been made in identifying loci controlling malic acid (malate) accumulation in tomato fruit (Sauvage et al., 2014). performed GWAS on metabolic traits in 163 tomato accessions using 5,995 SNPs and detected malate-associated loci on chromosomes 2 and 6 (Bauchet et al., 2017). analyzed a panel of ~300 tomato accessions using >10,000 SNPs and reported an association peak on chromosome 6 that included candidate genes annotated as malate transporters. Ye et al. (2017) integrated mGWAS, linkage mapping, and functional analyses to identify a major-effect locus, TFM6 (tomato fruit malate 6), on chromosome 6 that regulates fruit malate accumulation. Zhao et al. (2019) conducted a meta-GWAS analysis on 755 tomato materials, identifying malate-associated variant loci on chromosomes 1, 9, 11, and12 (Zhao et al., 2019). More recently, Gai et al. (2023) employed multiple LD-based and lead SNP–based association models across two environments and identified 15 high-confidence annotated genes, including TFM6 on chromosome 6; additional malate-associated SNPs were detected across several chromosomes depending on the environment and model (Gai et al., 2023).

In the present study, malic acid content was quantified in 200 tomato lines at the second-truss red-ripening stage. Using EMMAX, FaST-LMM, and GEMMA for association analysis, we identified six significant malic acid–associated SNP loci, all located on chromosome 6. This agreement with prior studies—particularly those implicating chromosome 6 and the TFM6 region - supports the robustness of our phenotyping and GWAS results and further highlights chromosome 6 as a key genomic region controlling malic acid accumulation in tomato fruit.

### 4.2 Malic acid candidate genes

The aluminum-activated malate transporter (ALMT) gene family plays a central role in malate transport across cellular membranes. In grape, VvALMT9 mediates vacuolar accumulation of malate and tartrate (De Angeli et al., 2013). In tomato, SlALMT4 and SlALMT5 have been shown to transport malate under electrophysiological assays, and overexpression of SlALMT5 increased malate and citrate levels in mature seeds (Sasaki et al., 2016). Consistent with earlier mapping of a major malate locus on chromosome 6 (Ye et al., 2017), our GWAS and LD analyses prioritized ALMT-related candidate genes in the same chromosomal region. Importantly, we identified specific SNP variants within ALMT-annotated genes that were significantly associated with fruit malic acid content, providing practical targets for marker-assisted selection in tomato breeding.

ATP-binding cassette (ABC) transporters mediate the active transport of diverse substrates, including organic acids and metal-associated complexes (Rea, 2007; Verrier et al., 2008). Several studies support a mechanistic link between ABC transporters and malate-related aluminum detoxification pathways. In Arabidopsis, the tonoplast-localized bacterial-type ABC transporter complex STAR1/ALS3 contributes to internal aluminum detoxification, and recent work indicates that malate secretion and subsequent transport of Al–malate complexes can contribute to internal tolerance (Yang et al., 2018); Fan et al., 2024). In addition, AtABCB14 has been reported as a malate transporter influencing stomatal responses (Lee et al., 2008). Building on these findings, our association and validation results showed that SNPs within the tomato *Sl*ABCB candidates (Solyc06g072950.3 and Solyc06g072960.1) were strongly correlated with malic acid content, suggesting that these ABCB genes may participate in malate transport or malate-associated cellular processes that influence fruit malate levels.

Increasing evidence also implicates NAC transcription factors in the regulation of organic acid metabolism and fruit acidity. For example, the apple NAC transcription factor MdNAC18.1 regulates organic acid accumulation and fruit acidity by activating downstream targets involved in malate-associated pathways (Liu et al., 2025). In tomato, NAC family members have been reported to respond rapidly to aluminum stress in root tissues (Jin et al., 2020; Feng et al., 2022), supporting a potential connection between stress-responsive transcriptional regulation and malate-related physiology. In this study, GWAS prioritized Solyc06g073050.2, annotated as the membrane-bound NAC transcription factor *Sl*NACMTF3. Although *Sl*NACMTF3 has been implicated in stress-responsive signaling in tomato (Bhattacharjee et al., 2017), our data indicate that sequence variation in *Sl*NACMTF3 was also associated with variation in fruit malate content. Further functional validation (e.g., expression profiling across fruit development, allele-specific expression, and gene editing) will be needed to determine whether *Sl*NACMTF3 influences malate accumulation directly, or indirectly through stress-related regulatory networks.

### 4.3 Development of molecular markers

Compared with conventional phenotype-based selection, marker-assisted selection (MAS) can improve breeding efficiency and accuracy by enabling early and reliable tracking of target alleles using molecular markers (Liu et al., 2021). PARMS is a fluorescence-based SNP genotyping method that uses universal fluorescent primers together with allele-specific primers and a common reverse primer to distinguish SNP alleles in a high-throughput format (Ye et al., 2001; Zhang et al., 2019). PARMS has been increasingly applied in crop breeding programs, including rice, tomato, and maize, due to its scalability and robust genotype calling (Zhao et al., 2022; He et al., 2023; Shi et al., 2023). A key advantage of PARMS is that it does not require gel electrophoresis of PCR products, thereby simplifying workflow while maintaining high accuracy (He et al., 2023). This feature makes PARMS well suited for large-scale screening in breeding pipelines.

In the present study, PARMS primers were developed for six SNP loci within candidate genes identified by GWAS. Genotyping showed a high call rate and clear clustering, and association with phenotypic data confirmed that these six loci were significantly correlated with malic acid content. Therefore, these SNP markers provide practical tools for rapid assessment and selection of fruit malic acid content in tomato breeding. To our knowledge, these PARMS markers represent a new application for malic acid–related loci in tomato, providing useful resources for marker-assisted improvement of fruit acidity.

## 5 Conclusion

In this study, a natural population consisting of 200 advanced-generation tomato inbred lines was used for GWAS of fruit malic acid content. Malic acid levels were quantified at the red-ripe stage, and GWAS identified a major association signal on chromosome 6, leading to the prioritization of five candidate genes: Solyc06g072910.2, Solyc06g072920.3, Solyc06g072950.3, Solyc06g072960.1, and Solyc06g073050.2. These genes were annotated as members of the ALMT (aluminum-activated malate transporter), ABCB (ATP-binding cassette transporter subfamily B), and NAC (NAC domain-containing transcription factor) families. Based on these candidates, molecular markers were developed for six SNP loci, and PARMS genotyping demonstrated clear genotype calls and strong correspondence between SNP genotypes and malic acid phenotypes. The significant associations observed indicate that these loci are promising markers for marker-assisted selection aimed at fruit malic acid content in tomato. Collectively, our results suggest that variation in ALMT, ABCB, and NAC gene families may contribute to the regulation of malic acid accumulation, providing a foundation for future functional studies to elucidate the underlying metabolic and regulatory mechanisms.

## Data Availability

The raw SNP dataset has been deposited in a public repository. The BioProject accession number is PRJCA059987, available at: https://bigd.big.ac.cn/gvm/getProjectDetail?Project=GVM001343. The associated SNP marker dataset is also available at: https://doi.org/10.6084/m9.figshare.31732636.
