# Supplementary material for: Identification of candidate genes for malic acid content in tomato fruits and development of associated SNP markers
Source: Front Plant Sci. 2026 Mar 24;17:1791734. doi: 10.3389/fpls.2026.1791734 (PMC13055536; doi:10.3389/fpls.2026.1791734)
Supplement: Supplementary file 1 [file DataSheet1.doc]

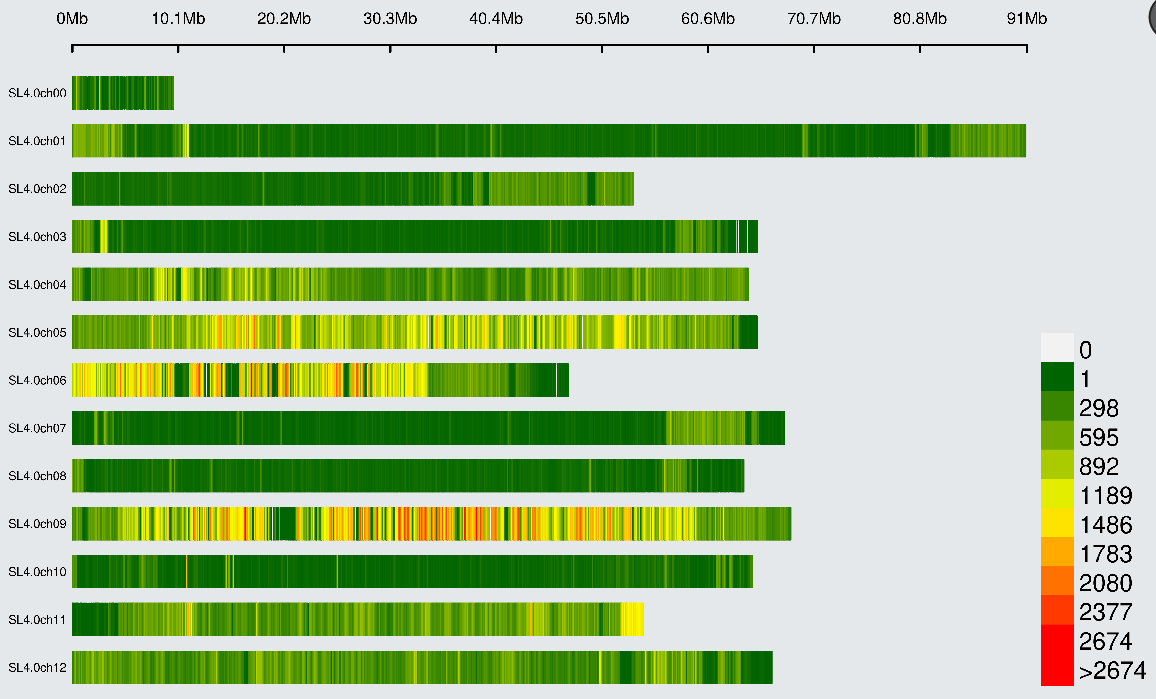


FIGURE S1 Distribution density of SNPs on each chromosome (0.1 Mb window)


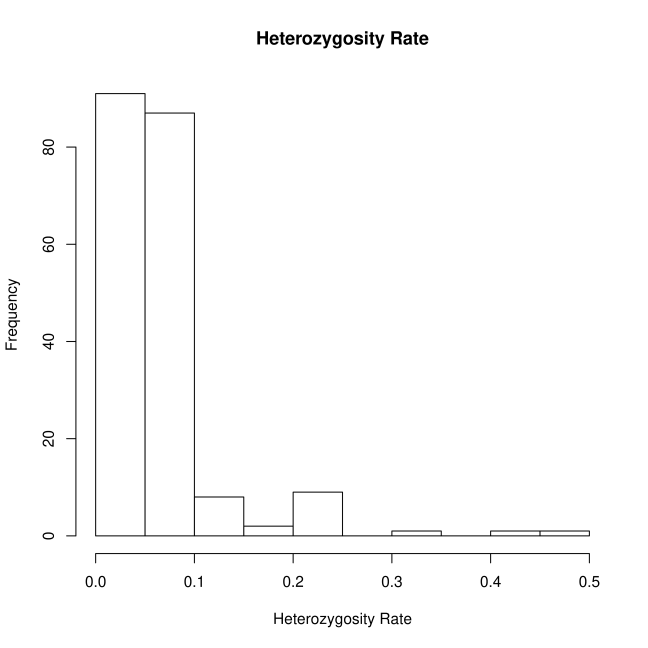


FIGURE S2 Frequency distribution histogram of individual heterozygosity rate

Horizontal axis: the heterozygosity of the sample (ranging from 0 to 1; the smaller the value, the higher the homozygosity). Vertical axis represents the frequency of the heterozygosity


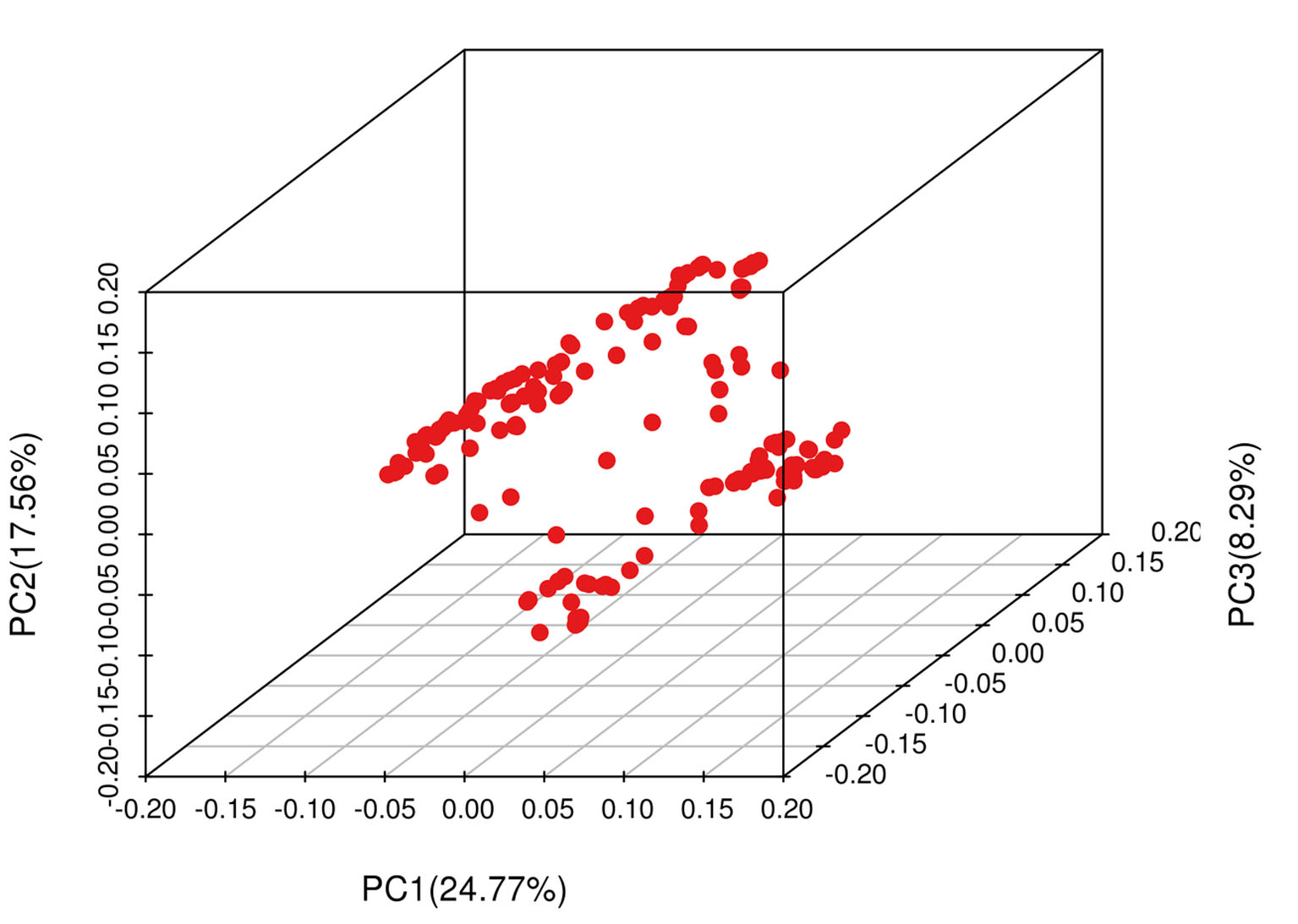


FIGURE S3 3D PCA clustering plot
